# Perception and Attitude about Child Sexual Abuse among Vietnamese School-Age Children

**DOI:** 10.3390/ijerph16203973

**Published:** 2019-10-18

**Authors:** Ha Ngoc Do, Hoa Quynh Thi Nguyen, Linh Thuy Thi Nguyen, Hiep Duy Nguyen, Thanh Phuong Bui, Nguyet Thanh Phan, Hang Thu Thi Do, Giang Hai Ha, Hai Thanh Phan, Trang Huyen Thi Nguyen, Anh Toan Ngo, Kiet Tuan Huy Pham, Bach Xuan Tran, Carl A. Latkin, Cyrus S. H. Ho, Roger C. M. Ho

**Affiliations:** 1 Youth Research Institute, Ho Chi Minh Communist Youth Union, Hanoi 100000, Vietnam; ngochayri@gmail.com; 2 Department of Research on Youth Culture and Lifestyle, Youth Research Institute, Ho Chi Minh Communist Youth Union, Hanoi 100000, Vietnam; hoaquynh1801@gmail.com; 3 Department of Research on Children’s Issues, Youth Research Institute, Ho Chi Minh Communist Youth Union, Hanoi 100000, Vietnam; linhthuy2401@gmail.com (L.T.T.N.); duyhiepk53xhh@gmail.com (H.D.N.); thanhxhh26@gmail.com (T.P.B.); 4 Department of Research on Youth’s Organizations and Youth Campaign, Youth Research Institute, Ho Chi Minh Communist Youth Union, Hanoi 100000, Vietnam; ptnguyet79@gmail.com; 5 Department of Research on Youth and Legal Issues, Youth Research Institute, Ho Chi Minh Communist Youth Union, Hanoi 100000, Vietnam; hanganhvtn@gmail.com; 6 Institute for Global Health Innovations, Duy Tan University, Da Nang 550000, Vietnam; haipt.ighi@gmail.com; 7 Center of Excellence in Pharmacoeconomics and Management, Nguyen Tat Thanh University, Ho Chi Minh City 700000, Vietnam; trang.coentt@gmail.com; 8 Institute for Preventive Medicine and Public Health, Hanoi Medical University, Hanoi 100000, Vietnam; ngotoananh85@gmail.com (A.T.N.); phamhuytuankiet_vkt@fpt.vn (K.T.H.P.); bach.ipmph@gmail.com (B.X.T.); 9 Bloomberg School of Public Health, Johns Hopkins University, Baltimore, MD 21205, USA; carl.latkin@jhu.edu; 10 Department of Psychological Medicine, National University Hospital, Singapore 119074, Singapore; cyrushosh@gmail.com; 11 Center of Excellence in Behavioral Medicine, Nguyen Tat Thanh University, Ho Chi Minh City 700000, Vietnam; pcmrhcm@nus.edu.sg; 12 Department of Psychological Medicine, Yong Loo Lin School of Medicine, National University of Singapore, Singapore 119228, Singapore; 13 Institute for Health Innovation and Technology (iHealthtech), National University of Singapore, Singapore 119077, Singapore

**Keywords:** children, sexual abuse, perception, attitude, knowledge, Vietnam

## Abstract

Child sexual abuse has become a significant public health concern in Vietnam in recent years, and the likelihood of being abused could be prevented by increasing the awareness of sexual abuse and self-protection skills among children. However, little is known about the perception and attitude of schoolchildren toward this issue in Vietnam. This study aimed to evaluate the knowledge and attitude of school-age children toward child sexual abuse and the risk factors affecting their knowledge and attitude. A cross-sectional study was conducted among 800 Vietnamese students from grades four to nine. Most of the respondents had insufficient knowledge of sexual abuse in children; teachers and strangers would not be perpetrators (57.9% and 74%); and schools and home were safe places (55.8% and 58.8%). Almost all participants disagreed with touching and non-touching actions, even from acquaintances (94.5% to 99.5%). Being female, older age, not living with family or relatives, and living in an urban setting were found to be positively associated with the right perception and attitude toward child sexual abuse. A sexuality education program should be officially applied at schools for children with the support of their parents to narrow the knowledge gap between different geographical locations and genders.

## 1. Introduction

Child sexual abuse (CSA) is a type of violence against children that has received significant concern worldwide [1,2]. The inconsistency in defining CSA has been noted since the 1970s [3,4,5,6,7], and this issue is one of the two main unsolved problems for this serious crime. The other problem are the different and unclear concepts of CSA such as “child sexual abuse”, “child sexual exploitation”, or “adverse sexual assault” [8]. Although there have been various definitions of CSA [9,10,11], we chose the definition of the World Health Organization (WHO). The WHO considers CSA as a sexual activity between a child and an adult or another child, in which a child “does not fully comprehend, is unable to give informed consent to, or for which the child is not developmentally prepared and cannot give consent, or that violates the laws or social taboos of society” [10]. 

Globally, the prevalence of child sexual abuse is 18% for girls and 7.6% for boys [12]. The World Health Organization (WHO) revealed that one in 13 men and one in five females suffered various forms of sexual violence whilst being a child [13]. The prevalence of child sexual abuse in Africa, Asia and Oceania, and Europe was 34.4%, 23.9%, and 9.2%, respectively [14]. Compared to these figures, the prevalence of child sexual abuse in Vietnam was lower, in the range of 2.6%–2.8% [15,16]. Another study confirmed that the prevalence of child sexual abuse by self-report in Taipei, Shanghai (China), and Hanoi (Vietnam) was 5.2%, 1.3%, and 0.5%, respectively [17]. However, it is difficult to identify the exact prevalence rates of child sexual abuse as this crime is often covered up and not reported [18]. Another explanation could be the inconsistency of the definition regarding child sexual abuse among researchers and organizations [19,20,21], which can lead to the difference in results from scientific articles, reviews, reports, or even policy and legacy. Moreover, it could be that the culture, child protection systems, legal systems, or healthcare systems in some countries are not sensitive to or developed enough to focus on this serious issue [22]. 

Even when child sexual abuse happens at a low rate, this crime not only causes short-term and long-term impacts on victims, but is also a worrisome burden to the family and society [1,16,23]. The personal consequences include an increased likelihood of suffering mental illness, psychiatric disorders [24], post-traumatic stress disorder [25,26], risk of suicide [27,28], substance use disorder [28], and a lower economic condition when compared with those who had not been abused [29]. Some studies have proven the existence of a vicious cycle when the abused child becomes the abuser among male perpetrators [30,31]. Other studies have cited economic impacts: child sexual abuse was reported to cause 0.39% loss of the gross domestic product for China [32], and the economic value of the disability-adjusted life years lost to child sexual abuse increased from 0.209% to 0.865% [33]. Thus, protecting children from child sexual abuse and instructing them to increase their awareness of the risks of this serious crime are necessary. However, children are vulnerable, dependent, and lack knowledge about danger and protection [34]. Increasing the awareness of children regarding sexual abuse could be one of the solutions to this serious issue. 

There have been several studies focusing on the perception and attitude toward child sexual abuse. Rural communities in Tanzania had accepting attitudes toward sexual violence [35] and parental responses showed that they had adequate knowledge regarding child sexual abuse [36,37]. However, most of the research has been aimed at investigating the perception and attitudes of men and women aged 18 years old and above [35,38,39], parents with children under 18 [40], or representatives of the socio-legal system (police investigators, magistrates, legal officers, and social officers) [41]. Meanwhile, children, who suffer the most regarding the consequences of this serious crime, had their opinions not given sufficient attention and even do not have enough cognition about the forms of CSA [42]. In particular, if the perpetrator was a family member due to embarrassment and shame to the family, these cases were sometimes covered up [43]. 

To our knowledge, in Vietnam, there has not been any study investigating the perception and attitude of school children (9–15 years old) regarding child sexual abuse. This is important information, especially in the context of Vietnam, where sex is culturally an under-discussed topic, non-professional parents do not provide sex education to their children, and schools do not offer enough information on sexual health (usually within a one-off 30-min session during a biology class) [44]. In Vietnam, the law on CSA is ambiguous, where some forms of sexual violence are still considered as inappropriate public behavior and people can be fined an administrative fee of only up to $13 [45]. This study, therefore, aims to explore what children know and how they think about child sexual abuse.

## 2. Materials and Methods 

### 2.1. Study Design and Sampling Procedure

A cross-sectional study was conducted from May to August 2017 in three provinces including Hanoi, Lao Cai, and Binh Thuan. In each province, we listed all of the public and private elementary and secondary schools, and randomly selected five elementary and five secondary schools by using computer software. Among the selected elementary schools, we chose children from grades four and five, while among the selected secondary schools, we chose all four grades (from grades six to nine). In each grade, we randomly singled out two or three classes and 10–15 students per class. The school students took part in the interview if they: (1) were aged from 10–16 years old; (2) were studying in the selected schools; and (3) agreed to participate in the study as well as had agreement from their teachers and their parents/caregivers. A total of 800 school-aged children were included in the study.

### 2.2. Data Measurements

Data were collected based on a self-administered questionnaire. Each questionnaire lasted 15 minutes. An interviewer introduced the purpose of the study briefly and carefully guided the participants in order to fill the questionnaire with the highest quality. A private room and seats were arranged to avoid discussions among students during the data collection. 

The collected information included the sociodemographic characteristics (gender, grade, living location, and people whom children were living with), perception, and attitude of school-aged children to child sexual abuse.

CSA involves several types of sexual activities toward children [2], however, for our objectives, we designed questions based on two types of sexual abuse: contact and non-contact abuse [46]. Regarding the children’s perception regarding child sexual abuse, we asked school children ten questions including: (1) express their opinions about possible victims of child sexual abuse (two items); (2) possible child sexual abuse offenders (three items); and (3) possible places and times of child sexual abuse (four items). Meanwhile, to reveal attitudes toward child sexual abuse, contact CSA was measured by asking “A teacher wants to touch the child’s body for a high score”, and non-contact CSA was measured by asking “Friends/neighbors want to tell stories related to touch, kiss, or hug”, and “Parents’ friends promise to us take out if we agree to see a picture related to sex”. A child will choose whether they agree or disagree with the above statement. Each correct answer for perception or attitude scored 1. The final scores of perception and attitude were transformed into a 10-point scale. 

### 2.3. Data Analysis

Stata 14.0 was used to analyze the data. A p-value under 0.05 represented the statistical significance. Chi-squared and Kruskal–Wallis tests were used to identify the differences in characteristics between male and female children. A multivariate Tobit regression was used to determine the predictors of perception and attitude scores. Cronbach’s alpha was used to assess the internal consistency of the questionnaire. The Cronbach’s alpha (α) of perception and attitude were 0.7537 and 0.7105, respectively. Stepwise forward selection strategies were used along with multivariate regressions at the threshold of 0.2 to construct the reduced model. 

### 2.4. Ethics Approval

Our participants were children aged from 9 to 15 years old. The researcher explained the study to their parents and the consent forms were signed by their parents. After enrolment, participants were assigned different identification codes (ID) and interviewers wrote the ID on each question sheet instead of the child’s identity. The interviewers had to sign a “security agreement” form as part of the employment contract. The protocol of this study was approved by the Institutional Review Boards (IRB) of the Youth Research Institute.

## 3. Results

Table 1 shows that most of the participants were female (58.6%), living with their father (90.5%), living with their mother (96.9%), and living in a rural area (43.7%). There were differences in grades, living with grandfather, grandmother, and other relatives among children in three locations (*p* < 0.05).

Table 2 reveals that the highest percentage of children agreeing with the following items “Children are at risk of sexual abuse at night and in places with a few people” (80.5%), “Only girls can be victims of child sexual abuse” (79.0%), and “Boys cannot be victims of child sexual abuse” (78.5%). The mean perception score was 6.8 (SD = 2.6).

Regarding attitude, Table 3 shows that the mean attitude score was 9.7 (SD = 1.0). No differences were found in each item and the total attitude score between males and females (*p* > 0.05).

Table 4 demonstrates that being female, being in a higher grade, and living with other people were positively correlated to the perception score, while children living in rural areas and mountainous regions had a lower score of perception when compared to urban areas (Coef. = −0.9; 95% CI = −1.4; −0.4; and Coef. = −1.7, 95% CI = −2.3; −1.2, respectively). Studying in a higher grade was also positively associated with having a higher attitude score.

## 4. Discussion

This study has provided some insights into the level of awareness and attitude of school-aged children on the issue of child sexual abuse. Although up to 99.5% of our participants did not agree with touching or non-physical contacts, they had insufficient knowledge of sexual abuse in children. The majority of them believed that boys could not be victims of sexual abuse, nor the perpetrators could be relatives, and more than half considered schools and their home a safe place. The results could contribute to enhancing the sex education programs at schools, in a family, or in the community to prevent this crime against children.

We found that the overwhelming majority (80%) of school children agreed that boys could not be victims of child sexual abuse. However, over 25 years, many studies have shown that boys have experienced sexual abuse or assault [12,13,47,48]. The awareness of male sexual abuse is incomplete [49], and research on male sexual abuse has lagged behind that of female sexual abuse [50]. This issue could be explained by the silence of survivors [51]. Several reasons may keep boys from revealing abuse including society as it does not encourage men to express feelings of helplessness and vulnerability [52,53], a fear of stigmatization [54], and that children do not fully understand what has happened [51].

Notably, three-fourths of school children believed that perpetrators would be strangers and could not be relatives or teachers. However, the research among female college students in Singapore showed that most perpetrators were neighbors or friends [55]. A similar result in a study conducted in Hong Kong confirmed that friends of the family or victims were the primary perpetrators (n = 96; 64.2%) [56]. In South Korea, peers were the foremost perpetrators with one-fifth (20.7%) of all research cases [57]. According to the result, the perception of Vietnamese schoolchildren to CSA was inadequate. Thus, a solution to this phenomenon is needed in further research.

Furthermore, more than half of the school children considered school and family a safe place (58.8% and 58.8%, respectively). However, the place where incidents of abuse occur are mainly at schools (24.9% among females from age 18–24 and 26.0% among males from age 18–24 confirmed this) [58]. Regarding peer sexual abuse, school settings were the place where sexual intercourse took place [57]. Moreover, a survey in 2014 revealed that home, which should be the safest place, turned out to be the most dangerous place for children [59]. 

The respondents also had the perception that a child was abused when they were alone, at night, and in a quiet place when few people were around. Almost all respondents disagreed with both touching and non-touching activities, even from an acquaintance. In Vietnam, the official sex education program has not been offered at schools, and Vietnamese parents are not eager to talk about sex as they consider sexuality to be sinful and taboo [44]. Therefore, the children have to find other sources of information such as the Internet or social networks. Nonetheless, some of them provided incorrect information, which can lead to misconceptions about sexual abuse to children. Therefore, unofficial information might lead to the failure to protect children from sexual abuse. 

Regarding the factors affected by the perception and attitude, our study identified four main factors: gender, age, living with other people, and living location. First, female school-aged children had better knowledge about child sexual abuse than their male school-age counterparts. This result is along the same line as others [60,61]. One explanation could be the concept that only females are victims of sexual abuse in Vietnamese society. Thus, they receive more concern from family, especially young parents (under 40 years old) who are more open to children asking and accessing sexuality information than older parents [44]. Our study also suggests that age is a protective factor for the proper perception of child sexual abuse. The increasing age leads to an increase in knowledge and experience in preventing child sexual abuse [35,37]. Notably, but not surprisingly, a child who does not live with parents or relatives have knowledge of child sexual abuse. Living with other people places children at a higher risk of being abused [62]; thus, this may force children to have to be equipped with the knowledge and skills to protect themselves. Last, but not least, children in urban areas have a better knowledge of child sexual abuse when compared with rural and mountainous areas. Factors related to those areas could explain this result. First, in Vietnam, the proportion of the population aged five and older who completed all education levels in urban settings was higher than that in rural settings [63]. The level of education could be a protective factor to the awareness of child sexual abuse. Second, a fear of stigma and the norms of keeping private matters private in many rural and mountainous areas may keep the perpetrator from being detected and prosecuted by authorities [64,65]. The result of this study could serve as preliminary evidence for designing further school-based education programs against sexual abuse among children in Vietnam. The difference in the knowledge about sexual abuse between schoolgirls and boys as well as a lack of awareness about the risks to those having close relationships with the children, even relatives or teachers, being perpetrators, as found by our study, should be taken into account when developing these education programs. Such consideration can be said to be even more important in the context of limited sexual/self-protection education program and knowledge among children in Vietnam. 

Some limitations should be considered. First, our research is a cross-sectional study; it would be challenging to conclude causality between the risk factors and outcomes. Second, the self-reported questionnaire might cause recall bias and social desirability response bias. Our interviewers were well-trained to recognize and minimize bias. Third, we did not investigate the knowledge of the schoolchildren about CSA, but focused only on perception and attitude. Finally, the participants were from three provinces in Vietnam. However, these three provinces were located in different geographic areas (urban, rural, and mountainous) with different social characteristics. Therefore, it may increase the generalization to the whole population. Primarily, the results of this study can still be used as evidence for identifying risk factors in child sexual abuse.

## 5. Conclusions

In summary, almost all of the school children in our study were aware of child sexual abuse and were non-supportive of touching or non-touching action. The findings of this study suggest a lack of official sexuality education programs for Vietnamese children, especially those living in rural or remote areas. This is an alarm for caregivers, people who work with children such as teachers, and policymakers to pay more attention on providing the children with accurate information on sexual abuse. This might help to increase the awareness of children to sexual abuse, and from that, the crime against children could be reduced and prevented.

## Figures and Tables

**Table 1:** Demographic characteristics of the children.

Characteristics	Male		Female		Total		*p*-Value
	n	%	n	%	n	%	
**Total**	331	41.4	469	58.6	800	100.0	
**Grade**							
4th	58	17.8	82	17.5	141	17.6	0.08
5th	67	20.2	74	15.8	141	17.6	
6th	57	17.2	61	13.0	118	14.8	
7th	56	16.9	91	19.4	147	18.4	
8th	49	14.8	71	15.1	120	15.0	
9th	43	13.0	90	19.2	133	16.6	
**People living with**							
Father	302	91.2	442	90.0	724	90.5	0.55
Mother	316	95.5	459	97.9	775	96.9	0.06
Grandfather	77	23.3	98	20.9	175	21.9	0.43
Grandmother	114	34.4	136	29.0	250	31.3	0.10
Other relatives	31	9.4	31	6.6	62	7.8	0.15
Other people (not family or relatives)	18	5.4	7	1.5	25	3.1	<0.01
**Living location**							
Urban	90	27.2	160	34.1	250	31.3	0.10
Rural	150	45.3	200	42.6	350	43.7	
Mountainous	91	27.5	109	23.3	200	25.0	

**Table 2:** Perception of school-age children regarding child sexual abuse (% having “agree” answers).

Characteristics	Male		Female		Total		*p*-Value
	n	%	n	%	n	%	
**Possible victims of child sexual abuse**							
Boys cannot be victims of child sexual abuse	243	73.4	385	82.1	628	78.5	<0.01
Only girls can be victims of child sexual abuse	246	74.3	386	82.3	632	79.0	0.02
**Possible child sexual abuse offenders**							
Teachers would not be child sexual abuse offenders	160	48.3	303	64.6	463	57.9	<0.01
Just be wary of strangers because they are a big risk that can harm children	206	62.2	344	73.4	550	68.8	<0.01
Relatives never commit child sexual abuse	222	67.1	370	78.9	592	74.0	<0.01
**Possible places and times of child sexual abuse**							
Children are at risk of sexual abuse at night and in places with few people	263	79.5	381	81.2	644	80.5	0.47
Residential areas, amusement parks, etc. are places where children are at risk of sexual abuse	183	55.3	261	55.7	444	55.5	0.39
School is a place where there is no possibility of sexual abuse of children	163	49.2	283	60.3	446	55.8	<0.01
Child sexual abuse cannot occur in the family	171	51.7	299	63.8	470	58.8	<0.01
	**Mean**	**SD**	**Mean**	**SD**	**Mean**	**SD**	
**Perception score (per 10 points)**	6.2	2.7	7.1	2.5	6.8	2.6	<0.01

**Table 3:** Attitude toward child sexual abuse among school-age children.

Characteristics	Male		Female		Total		*p*-Value
	n	%	n	%	n	%	
**A teacher wants to touch child’s body for a high score**							
Agree	3	0.9	1	0.2	4	0.5	0.17
Disagree	328	99.1	468	99.8	796	99.5	
**Friends/neighbors want to tell stories that related to touch, kiss, hug, etc.**							
Agree	21	6.3	23	4.9	44	5.5	0.38
Disagree	310	93.7	446	95.1	756	94.5	
**Parents’ friends promise to take out if agreeing to see the picture related to sex**							
Agree	10	3.0	11	2.4	21	2.6	0.56
Disagree	321	97.0	458	97.6	779	97.4	
	**Mean**	**SD**	**Mean**	**SD**	**Mean**	**SD**	
**Attitude score**	9.7	1.1	9.8	1.0	9.7	1.0	0.15

**Table 4:** Factors associated with perception and attitude toward child sexual abuse.

Characteristics	Perception about Child Sexual Abuse		Attitude toward Child Sexual Abuse	
	Coef.	95%CI	Coef.	95%CI
**Gender (Female vs. Male)**	0.8 ***	0.3; 1.2	1.5 *	−0.3; 3.3
**Grade (vs. 4th)**				
5th	1.1 ***	0.4; 1.8	3.0 **	0.3; 5.8
6th	1.5 ***	0.8; 2.2	5.1 ***	1.5; 8.6
7th	1.7 ***	1.0; 2.4		
8th	1.7 ***	0.9; 2.4		
9th	2.6 ***	1.9; 3.3		
**Living with other people (not family or relatives) (Yes vs. No)**	2.0 ***	0.9; 3.2		
**Living location (vs Urban)**				
Rural	−0.9 ***	−1.4; −0.4		
Mountainous	−1.7 ***	−2.3; −1.2	1.6	−0.6; 3.7

* *p* < 0.1; ** *p* < 0.05; *** *p* < 0.01.
